# Recent treatment and novel imaging studies evaluating treatment of internet gaming disorder: a narrative review

**DOI:** 10.3389/fpsyt.2024.1408560

**Published:** 2024-06-13

**Authors:** Rishi Sharma, Aviv M. Weinstein

**Affiliations:** ^1^ Department of Psychology, Ariel University, Ariel, Israel; ^2^ Behavioral Science, Ariel University, Ariel, Israel

**Keywords:** Internet gaming disorder, Internet addiction, cognitive behavioral therapy, pharmacological treatment, brain imaging

## Abstract

Internet Gaming Disorder (IGD) is an emerging public health concern; effective treatments are still under development. This mini-review focuses on summarizing the main scientific evidence from psychological, pharmacological, brain imaging, and emerging treatment approaches for IGD. We searched PubMed and Scopus databases using keywords related to IGD and treatment. Cognitive behavioral therapy (CBT) is the most extensively researched psychological treatment for IGD, supported by several randomized controlled trials (RCTs). Other promising approaches include mindfulness, relapse prevention, abstinence protocols, and family therapy. Pharmacological treatments like bupropion and escitalopram have shown benefits, especially when IGD is comorbid with conditions like major depressive disorder. However, the quality of evidence is moderate for psychological interventions but low to moderate for pharmacological approaches. Emerging treatments such as transcranial direct current stimulation (tDCS), repetitive transcranial magnetic stimulation (rTMS), and electro-acupuncture have demonstrated efficacy in reducing IGD symptoms and modulating brain activity. Brain imaging techniques like functional magnetic resonance imaging (fMRI) have provided insights into the neural mechanisms underlying IGD and treatment effects, although these studies lack randomized controlled designs. While multimodal approaches show promise, larger, well-designed RCTs are needed to establish effective IGD treatments.

## 1 Introduction

Internet Gaming Disorder (IGD) is listed for further study in the Fifth Edition of the Diagnostic and Statistical Manual of Mental Disorders (DSM-5) (1) and as an addictive disorder in the 11th revision of the International Statistical Classification of Diseases and Related Health Problems (ICD-11) (2). Despite the inclusion of IGD as a clinical diagnosis, there has been slow progress in the development of effective treatment for IGD. IGD is a growing public health concern, affecting both adults (0.3–2.4%) and 7.3% of adolescents (male, 6.8%> female, 1.3%) and globally 2.47% (1.46%–4.16%) (3, 4), while another scoping review reported that IGD prevalence varied from 0.21–57.50% in general populations, 3.20–91.00% in clinical populations, and 50.42–79.25% in populations undergoing intervention for severe cases (5). Adolescents experience hyperactivity, inattention, lower self-esteem, social support, and life satisfaction (6). Hyperactivity/inattention and self-esteem problems seem to be important for the development of IGD, and IGD could prospectively contribute to a deterioration of adolescent mental health (7). Young adults with IGD reported symptoms of depression, anxiety, and stress, lower life satisfaction and elevated motor and attentional impulsivity (8).

Previous reviews of treatment studies (9–12) have several limitations, including problems in the measurement of use and lack of randomized controlled clinical trials. Lampropoulo et al. (13) evaluated treatment interventions in children and adolescents and found that CBT is a widely used intervention; however, for cases with comorbidity, such as depression or attention-deficit/hyperactivity disorder (ADHD), pharmacological intervention proved effective. Danielsen et al. (14) examined treatment studies and proposed a positive impact of therapeutic interventions on gaming disorder, limited by small-study effects, potential publication bias, a restricted study sample, and a lack of standardization. Dong et al. (15) reviewed treatment studies, including established approaches like CBT and newer options like pharmacotherapies and non-invasive brain stimulation (NIBS). They have suggested that CBT is the most extensively researched treatment for IGD and advice cautious implementation of medication-based approaches. In this narrative review, we evaluate the impact of various treatments on IGD behavior, with a particular emphasis on brain imaging findings and recent developments in both psychological and non-invasive treatment options.

## 2 Methods

We conducted searches on PubMed/Medline and Scopus using the keywords ‘Internet Gaming Disorder’, ‘Online Gaming’, ‘Brain Imaging’, and ‘Treatment/Intervention’. This yielded 75 studies, out of which 22 were included (10 RCT, 5 new treatments, 7 brain imaging studies). We specifically focused on randomized controlled trials (RCTs) for psychological and pharmacological treatments for IGD. Both authors screened each citation independently, and discrepancies were addressed in the discussion.

## 3 Results

### 3.1 Psychological treatment- CBT interventions

Several studies (**Table 1**) support CBT as a treatment for IGD. CBT and bupropion were more effective than bupropion alone after 8 weeks of intervention (16). A 6-week study found that both CBT and counselling reduced IGD severity, with no significant difference between the two treatments (17). A single-session implicit training to avoid gaming cues reduced automatic approach tendencies (18). CBT and virtual reality therapy reduced gaming addiction scores (19). A 15-week CBT treatment was more effective than a waiting-list control group in reducing IGD symptoms (20). Additionally, addressing and improving life satisfaction as a secondary outcome measure in this study has been shown to enhance long-term treatment effects and prevent relapse in internet use disorders (21). Training with a stimulus-response-compatibility (SRC) was superior to a pseudo-modification group (22). Finally, a large multicenter RCT with at-risk adolescents found that CBT effectively reduced IGD and unspecified internet use disorder symptoms over 12 months without impacting incidence rates (23). Overall, CBT, medications, and targeted behavioral interventions show promise for treating IGD.

**Table 1:** Summary of findings: List of studies included psychological and pharmacological treatment for IGD.

Nr.	Study (Reference)	Sample (n)	Study Design	Diagnostic assessment	Intervention	Duration	Strength of evidence (GRADE)	Remark
Psychological treatment								
1.	Kim et al., (2012) (16)	65 depressed adolescents with excessive online gameplay (32 males and 33 females)	RCT	YIAS	CBT+ Bupropion; Bupropion only	8 weeks and follow-up after 4 weeks	Low Risk of Bias: Unclear Inconsistency: NA Indirectness: No Imprecision: Serious Publication Bias: Unclear	CBT, in combination with bupropion, may be effective for the treatment of depressed adolescents with online game addiction, particularly in reducing online gameplay and anxiety, as well as in improving life satisfaction.
2.	Li and Wang (2013) (17)	28 IGD participants (14 males and 14 females)	RCT	IAS	CBT	6 weeks	Low Risk of Bias: Unclear Inconsistency: NA Indirectness: No Imprecision: Serious Publication Bias: Unclear	CBT and basic counselling had similar treatment effects on IAS scores and OGCAS scores.
3.	Rabinovitz and Nagar (2015) (18)	38 IGD participants (19 males and 19 females)	RCT	MMORPG assessment	cognitive bias modification via the Approach Avoidance Task	A single session	Low Risk of Bias: Unclear Inconsistency: NA Indirectness: No Imprecision: Serious Publication Bias: Unclear	Single-session training decreased automatic action tendencies to approach gaming cues. These effects occurred outside subjective awareness. Approach bias retraining reduced subjective urges and intentions to play, as well as decreased game-seeking behavior.
4.	Park et al., (2016) (19)	24 IGD participants	RCT (prospective trial)	YIAS	CBT or Virtual Reality Therapy	4 weeks	Low Risk of Bias: Unclear Inconsistency: NA Indirectness: No Imprecision: Serious Publication Bias: Unclear	Both CBT and VRT showed reductions in YIAS scores.
5.	Wölfling et al. (2019) (20)	IGD; n = 72 or waiting list control (WLC) group (n = 71)	RCT	AICA	Short-term CBT	15 weekly group and up to 8 two-week individual sessions.	Moderate Risk of Bias: Low Inconsistency: NA Indirectness: No Imprecision: No Publication Bias: Unclear	50 of 72 men (69.4%) in the CBT group showed remission 17 of 71 men (23.9%) in the WLC group showed remission.
6.	He et al. (2021) (22)	IGD n=24 HC=24	RCT	DSM-5 IGD criteria	IGD participant: stimulus-response compatibility (SRC) HC group: pseudo-modification training.	4-Days	Low Risk of Bias: Unclear Inconsistency: NA Indirectness: No Imprecision: Serious Publication Bias: Unclear	The findings suggest IGD involves an approach bias toward gaming cues, which stimulus-response-conflict reverse training can significantly reduce, providing therapeutic benefits.
7.	Lindenberg et al., (2022) (23)	422 at-risk adolescents for IGD (229 females) CBT n=167 Control n=255	RCT	CSAS, a modified German videogame dependence scale	A manualized, CBT preventive group intervention	4 sessions	Moderate Risk of Bias: Low Inconsistency: NA Indirectness: No Imprecision: No Publication Bias: Unclear	The CBT (PROTECT) group showed a greater reduction in symptom severity of gaming disorder or unspecified internet use disorder.
Pharmacological treatment								
8.	Han and Renshaw (2012) (24)	57 males with excessive gaming and depression	RCT	YIAS	Bupropion + education (n=29); Placebo + educated (n=28)	8 weeks	Low Risk of Bias: Unclear Inconsistency: NA Indirectness: No Imprecision: Serious Publication Bias: Unclear	Bupropion treatment lowered cravings and online game time compared to placebo. These benefits lasted even after stopping the medication.
9.	Song et al., (2016) (25)	119 adolescents and adults with IGD	RCT	YIAS	Bupropion + Escitalopram; No treatment control	6 weeks	Moderate Risk of Bias: Unclear Inconsistency: No serious Inconsistency Indirectness: No Imprecision: No serious Imprecision Publication Bias: Unclear	Both bupropion and escitalopram were effective in treating and managing IGD symptoms. Bupropion appeared to be more effective than escitalopram in improving attention and impulsivity in IGD patients. Attention and impulsivity seem to be important for the management of IGD.
10.	Nam et al., (2017) (26)	30 adults with IGD and MDD: Bupropion (n=15) Escitalopram (n=15)	Double-blind RCT	YIAS	Bupropion SR or Escitalopram	12 weeks	Low Risk of Bias: Unclear Inconsistency: No serious Inconsistency Indirectness: No Imprecision: Serious Publication Bias: Unclear	IGD and MDD symptoms in both groups improved after 12 weeks of treatment

AICA, Assessment of Internet and Computer Game Addiction; CBT, Cognitive Behavioral Therapy; CBI, Craving behavioral intervention; CGAI, Computer Gaming Addiction Invention; OGAS, Online Game Addiction Scale; CIAS, Chen Internet Addiction Scale; IAS, Internet Addiction Scale; MMORPG, Massive Multiplayer Online Role Playing Gamers; SAS, Self-Rating Anxiety Scale; OGCAS, Online Game Cognitive Addiction Scale; YIAS, Young Internet Addiction Scale; MDD, Major Depressive Disorder; NA, Not Applicable.

GRADE Working Group grades of evidence.

High certainty: Further research is very unlikely to change our confidence in the estimate of effect.

Moderate certainty: Further research is likely to have an important impact on our confidence in the estimate of effect and may change the estimate.

Low certainty: Further research is very likely to have an important impact on our confidence in the estimate of effect and is likely to change the estimate.

### 3.2 Pharmacological treatment for comorbid IGD and psychiatric disorders

There is evidence for comorbidity of IGD with anxiety disorder and major depressive disorder (MDD). In a 12-week, double-blind RCT, 50 males with IGD and major depressive disorder (MDD) were assigned to bupropion + education or placebo + education groups (24) see **Table 1**. During active treatment, the bupropion group showed a reduction in Internet Addiction scores, gaming time and Depression Inventory (BDI) scores versus placebo. During the 4-week follow-up, bupropion’s benefits on gaming persisted, but depression recurred, suggesting it may temporarily improve comorbid depression and internet gaming disorder (24). A subsequent 6-week study among adolescents and adults with IGD compared the effects of bupropion and escitalopram to no medication, and both medication groups showed improvement in clinical symptoms versus no medication group, with bupropion being more effective in improving attention and impulsivity (25). In a 12-week, double-blind trial by Nam et al. (26) involving 30 patients with IGD and MDD, treatment with bupropion or escitalopram improved depressive and IGD symptoms in both groups, but bupropion showed greater efficacy in reducing impulsivity and attentional symptoms.

### 3.3 Emerging treatment approaches

#### 3.3.1 Transcranial direct current stimulation

Transcranial direct current stimulation (tDCS) targeting the dorsolateral prefrontal cortex (dlPFC) reduces substance use and addictive behaviors, possibly by enhancing inhibitory control over addiction-related triggers. Lee et al. (27) administered tDCS over the DLPFC to 15 online gamers 3 times a week for 4 weeks. They reported decreased weekly hours spent on games, and Internet Addiction Test scores restored the symmetry of DLPFC metabolism. Wu et al. (28) investigated this possibility in 33 males with IGD who underwent active tDCS (1.5 mA for 20 minutes) and sham treatment 1 week apart, randomly assigned, to evaluate inhibitory control over gaming-related distractors and pre- and post-stimulation cravings. Active tDCS reduced interference from gaming distractors but did not affect cue-induced craving. This study was limited by small size and lack of addiction behavior assessments for tDCS.

#### 3.3.2 Repetitive transcranial magnetic stimulation

Cuppone et al. (29) reported on a 21 young male university student with excessive online gaming activity who received high-frequency rTMS over the left dorsolateral prefrontal cortex (DLPFC), resulting in reduced addictive symptoms and improved executive control, suggesting rTMS may represent an effective treatment for IGD.

#### 3.3.3 Electro-acupuncture

Recent studies have shown efficacy in using electro-acupuncture (EA) to treat IGD. Yang et al. (30) studied 32 adolescents with IGD who were randomly assigned to receive either EA (16 participants) or 16 receiving CBT, and 16 healthy volunteers were included as a control group. All adolescents underwent a 45-day intervention. Barratt Impulsiveness Scale (BIS-11) scores, Young’s Internet Addiction Test (IAT), as well as the ratio of brain N-acetyl aspartate (NAA) to creatine (NAA/Cr) and choline (Cho) to creatine (Cho/Cr), were measured by magnetic resonance spectroscopy before and after intervention. Both EA and CBT groups exhibited a decrease in IAT and BIS-11 total scores after treatment. However, the NAA/Cr and Cho/Cr ratios improved in the EA group after treatment but not in the CBT group, indicating that EA might offer benefits over CBT regarding impulsivity control and neuronal protection for individuals with IGD. Peng et al. (31) used electropuncture (EA), psychological intervention (PI), and comprehensive intervention (CI) for the treatment of depression in Internet addiction disorder (IAD). The study involved 120 subjects diagnosed with IAD who were randomly assigned to EA, PI, or CI groups for 40 days. All three treatments effectively reduced scores on IAT, SDS, and HAMD. However, the CI was most effective, followed by EA, and then PI. RCTs conducted by Wu et al. (28) and Yang et al. (30) show promise for tDCS and electro-acupuncture, respectively, while in the prospective single-arm study by Lee et al. (27) the absence of a control group limits the conclusion about its effectiveness; Cuppone et al. (29) offer insights on rTMS for behavioral addiction but lacks generalizability due to the case study format and the absence of a control group.

## 4 Assessment of the quality of the evidence using GRADE method.

Quality of evidence assessments were carried out using Grading of Recommendations Assessment, Development and Evaluation (GRADE) approaches (32). GRADE assessed each study on risk of bias, inconsistency, indirectness, imprecision, and publication bias. An overall high, moderate, low rating was assigned to the body of evidence for each intervention (**Table 1**). Overall, the quality of evidence for treating IGD is moderate for psychological interventions (mainly CBT) but low to moderate for pharmacological approaches (bupropion and escitalopram). While two large, well-designed RCTs support CBT (20, 23), most other studies for both intervention types suffer from limitations like small sample sizes and potential bias.

## 5 Discussion

### 5.1 Psychological treatment for IGD

CBT is a robust and validated intervention for IGD, only 6 RCTs have evaluated CBT to treat IGD. Han et al. (33) found CBT superior to supportive therapy, with a 66.3% improvement rate and greater reduction in internet addiction, anxiety, impulsivity, and social avoidance. Others have used a more specific treatment for impulsivity; for example, Zheng et al. (34) compared the effects of a single rash impulsiveness intervention (SRC) or behavioral reward sensitivity intervention versus a combined intervention among IGD participants. They reported that the behavioral training of Go/No-go and SRC effectively improved rash impulsiveness and reward sensitivity in IGD, respectively. Still, the intervention effect of the combined training was better than that of the single training. André et al. (35) evaluated relapse prevention as an IGD treatment in an RCT with Swedish adolescents. Both the treatment and control groups showed lower game addiction scale scores at follow-up, but the treatment group had greater improvement compared with treatment as usual. Integrating CBT, motivation, and relapse prevention can also improve treatment for IGD. Sharma et al. (36) reported improvement in 33 individuals with IGD after a 10-session (8-week) intervention combining motivational enhancement, cognitive restructuring, behavioral techniques and relapse prevention. Abstinence is also considered as a potential treatment for IGD. King et al. (11) piloted a voluntary 84-hr abstinence protocol in 24 online gamers, including 9 with IGD. Brief voluntary abstinence successfully reduced gaming hours, maladaptive cognitions, and IGD symptoms. At the 28-day follow-up, 75% of the IGD group showed clinical improvement, and 63% had reduced maladaptive gaming cognitions. Li et al. (37) reported an 8-week mindfulness-based treatment for 30 adults with IGD or problematic gaming, compared to a support group control. Participants in mindfulness treatment had substantial reductions in IGD symptoms meeting DSM-5 criteria, craving, and maladaptive cognitions that were maintained at a 3-month follow-up. Li et al. (38) reported that changes in maladaptive gaming cognitions mediated the beneficial effects of mindfulness for IGD. These findings suggest mindfulness can be an effective approach for targeting cognitive and behavioral symptoms of IGD. Sakuma et al. (39) reported on an intensive 9-day therapeutic residential camp for 10 adolescent males with IGD in South Korea, which comprises psychotherapy, psychoeducational therapy, and CBT components. Months after the camp, participants showed reduced total gaming time, problem recognition related to IGD, and increased self-efficacy for positive change. Finally, Nielsen et al. (40) evaluated family therapy for IGD in 30 adolescents meeting at least 5 DSM-5 IGD criteria, randomized to 12 receiving family therapy or 18 receiving treatment as usual in Switzerland. Over one year, both groups showed decreased IGD prevalence and fewer criteria met, with family therapy outperforming treatment as usual. However, there was no effect on total gaming time. These findings suggest family-based interventions may be beneficial for reducing core IGD symptoms among adolescents beyond standard treatment, though impacts on actual gaming behaviors require further study.

#### 5.1.1 Brain imaging studies of treatment of IGD

Brain imaging techniques such as fMRI have provided valuable insights into the neural mechanism underlying IGD and the effects of various treatments. Multiple studies have utilized fMRI to evaluate cue reactivity and craving in individuals with IGD pre and post-interventions, such as craving behavioral intervention (CBI) and acupuncture (**Table 2**). Zhang et al. (41, 42) found that CBI reduced cue-induced craving, insula activation, and insula connectivity with regions involved in visual processing and attention. Wang et al. (43) reported that recovered individuals exhibited reduced brain activity in the anterior cingulate cortex and lentiform nucleus when exposed to gaming cues, suggesting improved control over cravings. Wang et al. (44) used multi-voxel pattern analysis to identify brain regions involved in successful CBI treatment outcomes. Liu et al. (45) found CBI improved connectivity within the default-mode network, salience and executive control networks in IGD. Regarding acupuncture, Wang et al. (46) observed a reduction in resting-state functional connectivity between the ventral rostral putamen and cortical regions after treatment, correlating with alleviated internet cravings. Another study by Wang et al. (47) showed that acupuncture modulated functional connectivity within the frontal-parietal network and between subcortical nuclei and this network, with symptom improvement correlating with decreased connectivity in specific regions. These imaging studies provide insights into the neural mechanism underlying IGD and the potential therapeutic effects of interventions like CBI and acupuncture. All these studies using fMRI to evaluate brain activity in individuals with IGD employed valid methodologies with control groups selected prior to the studies. However, a limitation is that these studies were not RCTs, which methodologically limits the generalizability and strength of their findings.

**Table 2:** Summary of imaging studies.

Nr.	Study & Year (Ref)	Study Design	Participants (n)	Imaging technique	Brain Region(s) analyzed	Major Finding	Methodological limitations
1.	Zhang et al. (2016) (41)	Interventional (pre-test post-test)	23 with IGD 17 with IGD	fMRI + Craving Behavioral Intervention (CBI) fMRI	Insula	CBI effectively reduced Internet Gaming Disorder (IGD) severity and cue-induced craving, possibly by changing the insula’s involvement in visual processing and attention	Non-RCT study could introduce selection bias and confounding factors, limiting strength of study.
2.	Zhang et al. (2018) (42)	Follow-up to Zhang et al. (2016)	Same participants as Zhang et al. (2016)	Craving Behavioral Intervention (CBI)	Ventral striatum-left inferior parietal lobule	IGD participants who underwent CBI showed a greater decrease in connectivity along with reduced addiction severity following the intervention	Lack of control limits isolating the effects of CBI.
3.	Wang et al. 2019 (43)	Observational (case-control)	154 with IGD 29 former IGD	fMRI	Anterior cingulate cortex (ACC) and lentiform nucleus	Individuals who no longer met the criteria for IGD after 1 year exhibited reduced brain activity when exposed to gaming cues	The lack of a control group and potential selection bias limit its conclusions.
4.	Wang et al. (2022) (44)	Observational (case-control)	40 with IGD 19 HC	fMRI with cue-reactivity task Multi-voxel pattern analysis (MVPA)	Right middle frontal gyrus, superior frontal gyrus, supra-marginal gyrus, anterior/posterior lobes of the cerebellum and left postcentral gyrus	MVPA was used to predict which IGD participants responded well to a 6-week CBI intervention	Study only includes young men, limiting the generalizability of findings to the broader IGD population
5.	Liu et al. (2021) (45)	Observational (case-control)	74 with IGD 41 HC	seed-based resting-state functional connectivity (rsFC)	Left angular gyrus and ventromedial prefrontal cortex (vmPFC)	IGD participants showed lower whole-brain connectivity compared to healthy controls. CBI improved connectivity within these networks	Self-reported data, prone to bias and inaccuracy.
6.	Wang et al.(2020) (46)	Interventional (pre-test post-test likely)	27 with IGD 30 HC	rs-fMRI + acupuncture treatment	Reward system (nucleus accumbens) and habit system (dorsolateral striatum); right ventral rostral putamen (VRP) and the left orbitofrontal cortex, premotor cortex, cerebellum, and right ventromedial prefrontal cortex	After 40 days of acupuncture, increased rs-FC in IGD participants was reduced, and the changes in rsFC positively correlated with alleviated internet cravings.	
7	Wang et al (2023) (47)	Interventional (pre-test post-test likely)	30 with IGD 30 HC	rs-fMRI + acupuncture treatment	Functional connectivity between subcortical nucleus (nucleus accumbens) and fronto-parietal network	Acupuncture improved communication between reward processing and cognitive control regions, potentially reducing cravings.	The study was conducted on adolescents, which may limit the generalizability of the findings to other age groups or populations

## 6 Consideration for treatment of IGD

Machado et al. (48) investigated gender differences among 115 adults (20 women, 95 men) seeking treatment for problematic internet use in Brazil. Women showed higher rates of psychiatric comorbidity like mood disorders, anxiety disorders, obsessive-compulsive disorder, post-traumatic stress disorder, and bulimia nervosa compared to men. Women exhibited greater severity in certain behavioral addictions, such as compulsive buying and disordered eating. Gender differences were observed in personality traits, with women scoring higher on impulsivity, novelty seeking, and self-transcendence.

## 7 Limitations and future directions

The study addresses a few limitations, including small sample sizes limiting generalizability, lack of long-term follow-up hindering evaluation of treatment durability, inconsistency in diagnostic criteria and assessment measures across studies, potential publication bias inflating treatment effects, and the need to consider co-occurring conditions like anxiety depression and ADHD that might confound results. Further limitations include cultural influences since most studies are carried out in Asian countries. In future research, we emphasize the importance of larger, diverse samples, longer follow-up periods, standardized diagnostic tools, comprehensive literature searches, and addressing co-morbidities. Public health initiatives involving policymakers, healthcare systems, educators and gaming industry stakeholders are critical for preventing and facilitating appropriate treatment access.

## 8 Ethical and legal issues

The treatment of IGD presents ethical concerns around informed consent, autonomy, and ensuring confidentiality. Legal issues like data protection and protecting minors’ rights also require attention. Pharmacological risk needs evaluation, and discrimination, stigma, and accessibility issues must be addressed.

## 9 Conclusion

IGD is a growing public health concern that demands attention and effective treatment strategies. This review has examined the current landscape of treatment approaches, including psychosocial interventions, pharmacological interventions, and multimodal strategies.

## Author contributions

RS: Writing – original draft, Writing – review & editing. AW: Writing – original draft, Writing – review & editing.
